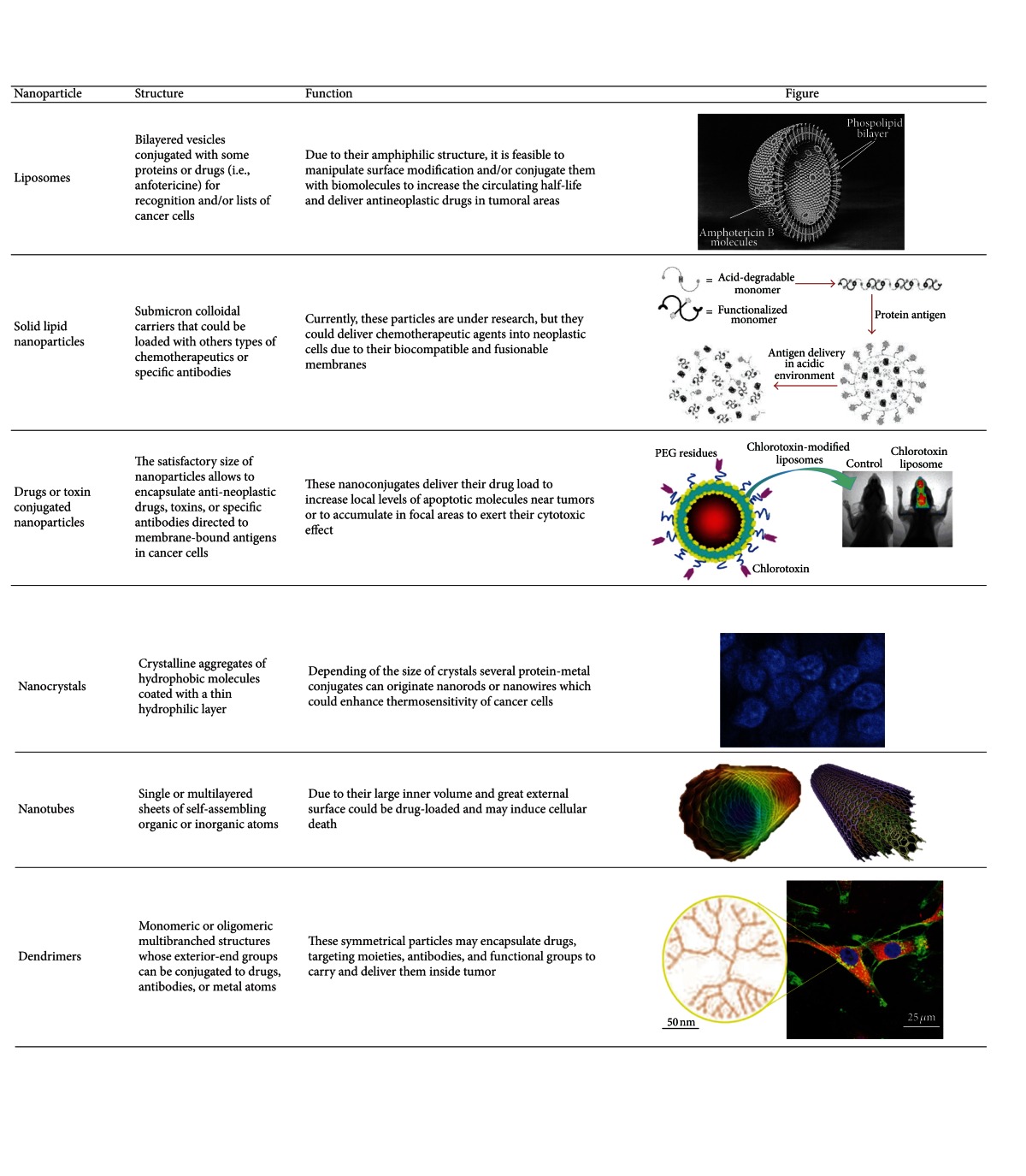# Application of Nanoparticles on Diagnosis and Therapy in Gliomas

**DOI:** 10.1155/2013/351031

**Published:** 2013-04-18

**Authors:** Norma Y. Hernández-Pedro, Edgar Rangel-López, Roxana Magaña-Maldonado, Verónica Pérez de la Cruz, Abel Santamaría del Angel, Benjamín Pineda, Julio Sotelo

**Affiliations:** ^1^Neuroimmunology and Neuro-Oncology Unit, National Institute of Neurology and Neurosurgery, 14269 Mexico City, DF, Mexico; ^2^Excitatory Amino Acids Laboratory, National Institute of Neurology and Neurosurgery, 3877 Mexico City, DF, Mexico

## Abstract

Glioblastoma multiforme (GBM) is one of the most deadly diseases that affect humans, and it is characterized by high resistance to chemotherapy and radiotherapy. Its median survival is only fourteen months, and this dramatic prognosis has stilled without changes during the last two decades; consequently GBM remains as an unsolved clinical problem. Therefore, alternative diagnostic and therapeutic approaches are needed for gliomas. Nanoparticles represent an innovative tool in research and therapies in GBM due to their capacity of self-assembly, small size, increased stability, biocompatibility, tumor-specific targeting using antibodies or ligands, encapsulation and delivery of antineoplastic drugs, and increasing the contact surface between cells and nanomaterials. The active targeting of nanoparticles through conjugation with cell surface markers could enhance the efficacy of nanoparticles for delivering several agents into the tumoral area while significantly reducing toxicity in living systems. Nanoparticles can exploit some biological pathways to achieve specific delivery to cellular and intracellular targets, including transport across the blood-brain barrier, which many anticancer drugs cannot bypass. This review addresses the advancements of nanoparticles in drug delivery, imaging, diagnosis, and therapy in gliomas. The mechanisms of action, potential effects, and therapeutic results of these systems and their future applications in GBM are discussed.

## 1. Introduction

Cancer is the most common cause of death in many countries. Central nervous system (CNS) tumors are an important cause of morbidity and mortality worldwide. It was estimated that 22,340 new cases of primary malignant brain and CNS tumors were diagnosed in the United States in 2011. Approximately 3,000 of them were new cases in childhood whereas about half of all CNS tumors were malignant in adults [1]. The distribution of CNS tumors shows that approximately 60% of these tumors have the typical glioblastoma histopathology [2]. Glioblastoma multiforme (GBM) comprises a heterogeneous group of neoplasms that differ in their location within the CNS; it is responsible for the 51% of all primary gliomas in adults and represents the second cause of cancer death in adults less than 35 years old [3]. Despite advances in diagnosis and treatment of GBM, their prognosis, incidence, and mortality rates remain poor.

Conventional treatment for malignant gliomas includes the use of chemotherapeutic drugs, radiotherapy, and interventional surgery [4]. However, both chemotherapy and radiotherapy give inconsistent results in terms of prolonging survival and response to treatment [5]. The median survival for GBM in patients subjected to the conventional multimodal therapies is 14.6 months, and the progression-free survival for recurrent GBM is less than 24 weeks [6, 7]. The conventional treatment for GBM shows some drawbacks that limit its potential use in therapy such as neurotoxicity, lack of specificity, poor drug accumulation in tumors, and severe side effects. Also, the blood-brain barrier (BBB) plays an important role limiting strategies of therapy, because several drugs have little or no solubility to cross this physical barrier.

Many approaches have been used to treat gliomas; however all of them have failed in modifying the prognostic and quality of life of patients suffering this devastating disease in the last decade. As the nanotechnology has expanded its application to biomedicine and biomedical areas, nanotoxicology has emerged to elucidate the relationship of the physical and chemical properties (size, shape, surface chemistry, composition, and aggregation) of nanostructures with induction of toxic biological responses [8]. Because these structures are small sized (less 100 nm), simple performed, fast and cheap in cost, they have been widely used in cytotoxic *in vitro* studies [9, 10]. Recently, nanotechnology is considered as a new tool for its application in diagnosis and treatment of malignant gliomas. Nanotechnology has revolutionized the conventional way in which gliomas therapy, diagnosis, and treatment are achieved mainly due to recent advances in material engineering, drug availability, and the advantage of targeting cancer cells, simply due to being accumulated and entrapped in cancer cells. This review is therefore primarily devoted to the current approaches used in imaging and treatment of gliomas. In addition, we present a brief description of the most common materials used in the design, composition, structure, and drug delivery systems by nanoparticles.

## 2. Use of Nanoparticles in Gliomas Diagnosis

In the imaging field, the development of nanoparticles as contrast agents has allowed obtaining detailed cellular and molecular imaging, monitoring drug delivery specifically to tumoral areas, and providing data for efficient surgical removal of solid tumors [11, 12].

Positron Emission Tomography (PET) is a well-established imaging modality that uses signals emitted by positron-emitting radiotracers to construct images about the distribution of the tracer *in vivo* [13, 14]. PET has provided valuable biophysiological information on various central nervous system disorders. In brain tumors, different radiotracers have been applied in PET studies to evaluate tumor blood flow and metabolism, as well as to detect tumors. Radiotracers such as 18F-labeled fluorodeoxyglucose ([18F]-*α*-methyl-tyrosine, L- and D-S-(3-[18F] fluoropropyl) homocysteine have been used for the PET imaging of tumors, but L- and D-S-(3-[18F] fluoropropyl)homocysteine biodegradation products generate a high background signal in the tissues [15]. In contrast, [2-18F]-2-deoxy-fluoro-D-glucose (FDG) has been the most frequently used marker for the evaluation of glucose metabolism in brain tumors. However, the utility of FDG-PET imaging for detection of brain cancer is controversial due to the small differences in rate of glucose utilization between normal brain and brain malignance (the FDG uptake is usually similar to that in normal white matter), and FDG-PET is effective in differentiating recurrent tumor from radiation necrosis for high-grade tumors, but it has limited value in defining the extent of tumor involvement and recurrence of low-grade lesions [16, 17].

**Figure 1:**
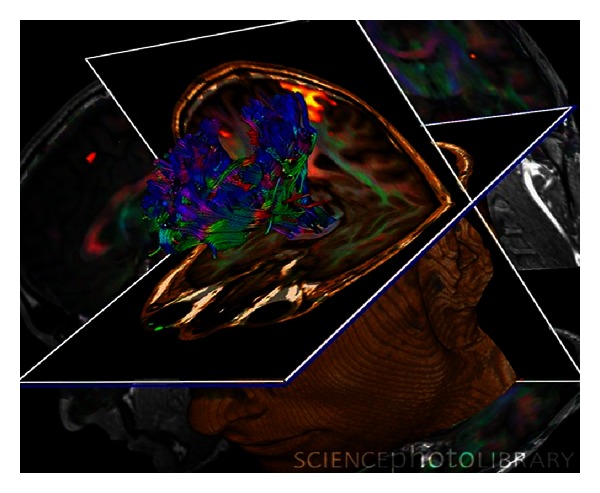
Brain tumour. Coloured 3D diffusion tensor imaging (DTI) and magnetic resonance imaging (MRI) scans of the brain of a 29-year-old with a low-grade glioma in the left frontal lobe. A DTI scan shows the bundles of white matter nerve fibers and is being used here for presurgical planning. The fibers transmit nerve signals between brain regions and between the brain and the spinal cord. A glioma arises from glial cells; nervous system supports cells. DTI scans show the diffusion of water along white matter fibers, allowing their orientations and the connections between brain regions to be mapped.

On the other hand, magnetic resonance imaging (MRI) is a widely accepted modality for providing anatomical information and high spatial-resolution anatomic images primarily based on contrast derived from the tissue-relaxation parameters T(1)- and T(2)*-weighted sequences. MRI is capable of visualizing various intracranial lesions and detecting the correlation between the major white matter fiber bundle and glioma lesions. The biggest advantage of a brain MRI is that it provides a good anatomical background without bone artifacts, and it is also capable of exhibiting the panorama and three-dimensional location of the tumor [18]. Nowadays, a novel neuroimaging modality has been developed for patients with brain tumors named functional magnetic resonance imaging (fMRI), which allows to obtain not only noninvasive measurements, localization, and lateralization of specific brain activation areas, but also the possibility to evaluate motor and speech functions, helping in the selection of the most appropriate, sparing treatment, and function-preserving surgery. However, fMRI cannot be considered as a fully established modality of diagnostic neuroimaging due to the lack of guidelines of the responsible medical associations as well as the lack of medical certification of important hardware and software components [19] (Figure 1).

To overcome these limitations, there are significant efforts in developing alternative imaging methods that are capable of enhancing the signal or generating bright and positive contrast [20, 21]. Some nanoparticles such as liposomal conjugates are known to accumulate in tumors due to the enhanced permeability of tumor blood vessels and the retention effect [22]. However, it still may not be possible to accurately localize an area of increased activity using PET images alone because of the absence of identifiable anatomic structures in nonrigid tissues, such as abdomen or brain [23, 24]. The greatest advantage of performing combined MRI compatible with PET scanners (PET/MRI) not only is to reduce radiation exposure, but also should theoretically be possible to obtain “perfect” spatial records of molecular/functional PET and anatomic/functional MRI studies [25, 26].

The use of macromolecular agents based on dendrigraft poly-L-lysines (DGLs), using chlorotoxin (CTx) as a tumor-specific ligand, has been explored with promising results in the field of clinical diagnosis of brain tumors using MRI studies, where it has been showed that the signal enhancement of mice treated with CTx-modified contrast reached peak level at 5 min for both glioma and liver tumor, significantly higher than unmodified counterpart. Most importantly, the signal enhancement of CTx-modified contrast agent is maintained much longer when it was compared to controls [27]. Recently, Veiseh et al. developed a nanovector comprised of a superparamagnetic iron oxide nanoparticle core coated with polyethylene-glycol- (PEG-)grafted chitosan and polyethylenimine (PEI). The functionality of the construct was achieved with short interfering RNA (siRNA) and the tumor-targeting peptide, chlorotoxin (CTx), to improve tumor specificity and potency. Receptor-mediated cellular internalization of nanovectors and the consequent gene knockdown through targeted siRNA delivery and the specific contrast of brain tumor cells were confirmed by flow cytometry, quantitative RT-PCR, fluorescence microscopy, and MRI studies [28]. This finding is especially important for tumors such as glioma which is known hard to be diagnosed due to the presence of BBB.

Magnetic nanoparticles (MNPs) represent a promising nanomaterial for the targeted therapy and imaging of malignant brain tumors. Conjugation of peptides or antibodies to the surface of MNPs allows direct targeting of the tumor cell surface and potential disruption of active signaling pathways present in tumoral cells [29]. It is known that magnetic nanoparticles also exhibit a higher longitudinal relaxivity, providing intrinsic signal enhancement on T1-weighted images [30]. Varieties of magnetic nanoparticles have been introduced as contrast agents for MRI and molecular imaging probes because of their super ability in shortening transverse relaxation times in T1- and T(2)*-weighted images, which leads to a strong decrease in signal intensity of target organs or so-called “negative contrast” on conventional T(2)*-weighted images.

The arginine-glycine-aspartic acid (RGD) sequence is currently the basic module for a variety of RGD-containing peptides which display preferential binding to *α* and *β*_3_ integrins, which play a key role in tumor angiogenesis and metastasis and were not detectable in normal blood vessels [31]. This probe could detect the tumor location with fluorescence imaging and assess the tumor-targeting efficacy of probe with radioactive analysis [32]. It has been proposed that the imaging techniques PET and MRI will greatly benefit from the use of bifunctional nanoprobe conjugates, such as polyaspartic-acid- (PASP-) coated iron oxide (IO) nanoparticles conjugated with cyclic RGD peptides and the macrocyclic chelating agent 1,4,7,10-tetraazacyclododecane-N, N′, N′′, N′′′-tetraacetic acid (DOTA) for integrin *α*_v_ *β*_3_ recognition. A displacement competitive binding assay indicates that DOTA-IO-RGD conjugates bound specifically to integrin *α*_v_ *β*_3_ *in vitro*. Small-animal PET and T2-weighted MRI showed integrin-specific delivery of conjugated RGD-PASP-IO nanoparticles and prominent reticuloendothelial system uptake. This bifunctional imaging approach may allow for earlier tumor detection with a high degree of accuracy [33–35].

### 2.1. Iron Magnetic Nanoparticles Auxiliary on Diagnosis

**Figure 2:**
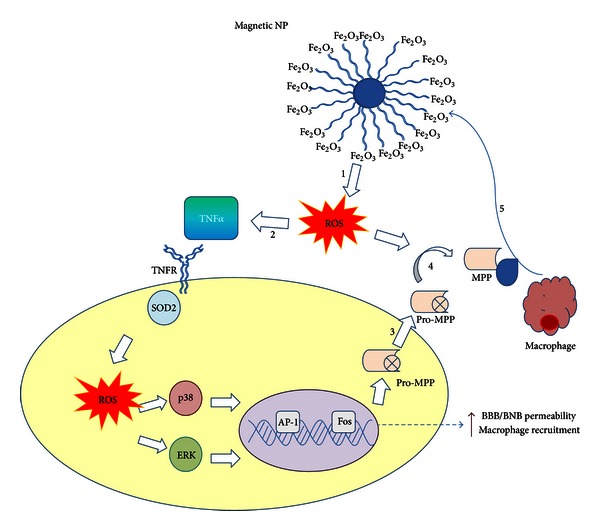
Proposed mechanism by Shubayev et al. [40] for MNP-induced macrophage recruitment into neuronal tissues. (1) Exposure to cytotoxic MNPs stimulated the formation of ROS in resident cells. (2) ROS promotes expression and release of proinflammatory cytokines, such as TNF-*α*. Through its two receptors (TNFR), TNF-*α* activates p38 and ERK mitogen-activated protein kinases pathways to (3) induce the expression of matrix metalloproteinases (MMPs) in its inactive, pro-MMP form. In addition, (4) ROS can directly promote MMP activation from proform. MMPs are the only enzymes in the body capable of degrading blood-brain and blood-nerve barriers (BBB/BNB), which (5) promotes infiltration of circulating macrophages (mΦ) into neuronal tissues. MNP size and surface chemistry determine the mechanisms and the target cells of MNP internalization, as well as extent of neurotoxicity of MNPs (the figure is taken and modified from [40]).

Over the past two decades, magnetic iron oxide nanoparticles (MPIOs) have been subject of extensive studies as an important class of MRI contrast agents for medical imaging [36, 37]. An MPIO, in general, is composed of maghemite (Fe_2_O_3_, *γ*-Fe_2_O_3_) or magnetite crystals less than 20 nm in diameter. These nanocrystals contain thousands of Fe atoms and approach saturation magnetization under a magnetic field typical for MRI [38]. In some *in vivo* studies it has been reported that absorption of these particles can occur through interactions with biological components such as proteins and cells; afterwards, they can distribute into various organs where they may remain in the same nanostructure or become metabolized [39]. It is known that MPIOs causes toxicity through the production of an excess of reactive oxygen species (ROS), including free radicals such as the superoxide anion, hydroxyl radicals, and the nonradical hydrogen peroxide. High ROS levels can damage cells by peroxidizing lipids, disrupting DNA, modulating gene transcription, altering proteins, and resulting in decline of physiological function and cell apoptosis/death [40] (Figure 2).

Superparamagnetic iron oxide particles (SPIOs) enhance contrast in MRI, which allows clinicians to monitor anatomical, physiological, and molecular changes during the evolution of a disease or treatment. Following intravenous injection, these nanoparticles accumulate in macrophages residing in the liver, bone marrow, and spleen, as well as tumors and sites of inflammation [41]. These particles are rapidly internalized by the mononuclear phagocytic system; consequently, they have been used in models of cell migration and homing in C6 models *in vivo* [42]. However the current applications for SPIO nanoparticles are limited because they have an average diameter of 80 nm in size, and it has shown relatively low toxicity in some *in vivo* applications [43–46]. Because SPIOs have a better resolution in MRI than conventional imaging, some changes in their structure has been developed to improve their diameter and diminish the adverse effects. Recently, these particles have been modified in ultrasmall superparamagnetic iron oxide (USPIO) particles which have diameters less than 50 nm and a longer half-life in the circulation system, allowing inclusively the labeling of macrophages migrating to remote areas [47].

Other nanoparticles composed of iron are the monocrystalline iron oxide nanoparticles (MIONs), which are nanoconjugates that permit accurate delineation of tumor margins which lead to an increase long-lasting signal of the tumor in T1-weighted sequences. In animal models, they constitute a contrast agent that is taken up by endocytosis by malignant glioma cells [48, 49]. The use of MIONs is a promising strategy to avoid surgically induced intracranial contrast enhancement, which is known to be a potential source of error in intraoperative MRI imaging of patients [50]. Currently, they have been used in animal models.

Manganese oxide nanoparticles have shown a prominent MRI T1 contrast using a U87MG glioblastoma xenograft model, and it has been confirmed that the particles can accumulate efficiently in tumor area to induce effective T1 signal alteration [51]. Additionally, pH-sensitive poly(lactic-co-glycolic acid) (PLGA)-encapsulated manganese oxide (MnO) nanocrystals have shown an excellent bright contrast on MRI following endocytosis of nanoparticles into the low pH compartments within the cells. Subsequently, these particles are degraded, and MnO dissolves to release Mn^2+^ causing the cells to appear bright on MRI. The magnitude of the change in MRI properties is as high as 35-fold, making it the most dynamic MRI contrast agent reported. Possible applications of these MnO particles include slow release of Mn^2+^, tumor targeting, and confirmation of cell uptake [52].

Gadolinium [Gd(H_2_O)_8_]^3+^ is the contrast agent mainly used in magnetic resonance imaging. However, it is known that high levels of gadolinium *in vivo* cause toxicity; therefore it requires the metal to be complexed by strong organic chelators. Gadolinium III (Gd III) is a highly paramagnetic complex with seven unpaired electrons, which have a strong impact on the relaxation of influenced water protons. Advances in colloidal nanocrystal synthesis have led to the development of ultrasmall crystals of gadolinium oxide (US-Gd_2_O_3_), with 2-3 nm in diameter, the smallest and the densest of all Gd-containing nanoparticles. Each nanocrystal can generate signal contrast of several orders of magnitude higher than a gadolinium chelate. Currently, US-Gd_2_O_3_ has been successfully used to label glioma cells GL-261 from localization and visualization *in vivo* using MRI. Because very high amounts of Gd are efficiently internalized and retained into the cells, it has been possible to detect the bright in T1-weighted MRI images [53]. The properties of the gadolinium-based particles give promising opening to a particle-assisted in imaging field.

Ultrashort echo time (UTE) imaging is able to track materials with extremely short T(2)*-weighted and very fast signal decay [54, 55]. With very short echo time (TE), typically below 0.1 milliseconds, UTE imaging allows signal acquisition with little T(2)*-weighted influence. The use of UTE imaging has allowed obtaining positive contrast imaging of U87MG human glioblastoma cells targeted with iron nanoparticles (IONPs) conjugated with a small RGD sequence, which has a high affinity to bound to cells overexpressing *α*_v_ *β*_3_ integrin such as ovarian carcinomas, breast carcinomas, gliomas, and other solid tumors [56–59]. A high concentration of RGD-containing probes must accumulate to overcome the limited sensitivity for the detection of contrast media. Therefore, UTE imaging may open the opportunity for the applications of magnetic nanoparticles with a strong T1 effect but also extremely short T(2)*-weighted [60].

Besides PET and MRI, fluorophores have been used in imaging applications. However, their use has been limited by poor quantum yield, poor tissue penetration of the excitatory light, and excessive tissue autofluorescence. The use of inorganic fluorescent particles that offer a high quantum yield is frequently limited due in part to the toxicity of the particles.

### 2.2. Nanoshells and Quantum Dots

Metal nanoshells and quantum dots are complexes that have shown good resolution in glioma imaging. Metal nanoshells are composed of a silica core surrounded by a thin metal shell or ultrathin coating of silver or gold [61]. These nanoparticles can be produced to absorb or scatter light, depending on the relative dimensions of the core size and shell thickness [62, 63]. Nanoshells have been used most commonly to treat murine gliomas. However, gold nanoshells have been used as contrast agents in optical imaging [64, 65], showing that these agents can both increase the surrounding water proton signals in the T1-weighted image and reduce the signal in T2-weighted images. Also, these nanoparticles exhibit strong absorption in the range of 600–800 nm, and their optical properties are strongly dependent upon the thickness of the gold-silver alloy shell. The intravenous administration of gold nanoshells has resulted in limited tumor accumulation, which represents a major challenge for contrast agents in optical imaging [64, 65]. Thus, these nanoshells have the potential to be utilized for tumor cell ablation due to physical characteristics (i.e., size, structure, and core), which when they are irradiated using laser light, they produce localized heat sufficient to damage tumor cells, ensuring a minimal thermal injury to the healthy tissue surrounding [66, 67].

Other nanoparticles which are now under extensive research are nanoshell conjugates of luminescent rare-earth-doped sodium ytterium fluoride (NaYF_4_), which are nanocrystalline infrared-to-visible upconversion phosphors, ytterbium (Yb), and erbium (Er) codoped NaYF_4_. These nanoparticles could be complexed with human serum albumin to originate water-dispersible nanoparticles, which could act as promising upconverting fluorescence labels when they are conjugated with cyclic arginine-glycine-aspartic acid (cRGD) sequence, specifically targeting both human glioblastoma cell lines and melanoma cells overexpressing *α*_v_ *β*_3_ integrin receptors. These characteristics offer an appropriate tool for targeted imaging of focal diseases [68, 69]. Rare-earth-doped nanoparticles utilize near-infrared upconversion, and they have been used to overcome the optical limitations of traditional fluorophores, but currently they are not typically suitable for biological application due to their insolubility in aqueous solution, lack of functional surface groups for conjugation with certain biomolecules, and potential cytotoxicity.

Quantum dots (QDs) are based on semiconductor compounds consisting of a cadmium-based core surrounded by an inert layer of metallic shell [70, 71]. Similar to gold nanoshells, quantum dots have excellent optical properties that are dependent on particle size. The tunable optical properties of these agents have primarily been used in preclinical optical imaging for a variety of cancer applications, including cellular and molecular imaging of brain tumors, including gliomas [72–74].

Near-infrared QDs composed of Cd(NO3)2, Hg(NO3)2, NaHTe (CdHgTe, CdTeSe/CdS), and a thiol group as stabilizer in gelatin solution are newly emerged as inorganic fluorescent probes. They provide several advantages over organic fluorophores for biological imaging, including broad excitation spectra coupled with narrow, tunable emission spectra and high resistance to photobleaching [75]. Lately, QDs have been used as excellent alternatives of traditional dyes in many fluorescence-based bioanalytical techniques [69, 76]. They exhibited strong fluorescence ranging from 580 to 800 nm that could be tuned by molar ratios of Hg^2++^ and gelatin. Compared with bare CdHgTe QDs, the photostability of this compact complex nanostructure is remarkably improved. The fluorescence of CdHgTe/gelatin nanospheres was much more resistant to the interference from certain endogenous biomolecules such as human serum albumin, transferrin, and hemoglobin [77].

In glioma cell cultures, nanospheres were small enough to be taken up by cells, and the fluorescence of QDs was not quenched inside the cells. Moreover, no morphological change of the cells was observed, indicating that the nanospheres were biocompatible. Some *in vivo* studies have shown that the CdHgTe/gelatin nanospheres are immediately distributed to all over the vessels by blood circulation after injection. A network of blood vessels could be distinguished in fluorescence images, and the dynamic changes of nanospheres in the superficial vessels were clearly observed [78]. Although, these particles could be used as promising nanocarriers for proteins, DNA, and small molecules in the research of *in situ*, real time monitoring of drug release and therapy studies in the near future.

In Table 1 are summarized some of the most applied nanoparticles in diagnosis of gliomas.

## 3. Nanoparticles as Therapeutics for Brain Tumors

Despite considerable advancements in therapy of malignant gliomas in the last years, treatment outcomes are mostly unsatisfactory. A promising way to bypass these impairments and to elicit the specific delivering of drugs to treat tumors within the CNS is the employment of biodegradable polymeric NPs, which can be loaded with different chemotherapeutic drugs to induce selective toxicity, and additionally, modulate cellular and humoral immune responses when looking for a specific immune response against tumoral cells [79]. A wide variety of NPs have been designed, each one with particular properties (certain size, shape, and composition) in a scale of strategies such as conjugated antigens, which are recognized by specific receptors [80]; antigens encapsulated within NPs, which offer the ability to protect the antigen from degradation; labeled NPs, which are also recognized by specific receptors and allow an effective tracking of their migration; and the use of NPs as vehicles for specific delivery of chemotherapeutic drugs [81, 82]. Some of the more representative nanoparticles used as carriers in the treatment of gliomas are described below.

### 3.1. Lipid Carriers

Liposomes are concentric bilayered vesicles surrounded by a phospholipid membrane. They are related to micelles which are generally composed by hydrophilic and hydrophobic regions. The amphiphilic nature of liposomes, their facility of surface modification, and a good biocompatibility profile make them an appealing solution for increasing the circulating half-life of proteins and peptides. They may contain hydrophilic compounds, which remain encapsulated in the aqueous interior, which may escape encapsulation through diffusion out of the phospholipid membrane. Liposomes can be designed to adhere to cellular membranes to deliver a drug payload or simply transfer drugs through endocytosis [83–86]. *In vitro* and *in vivo* experiments have indicated that the activity of a range of drugs or their active metabolites may be enhanced by their encapsulation in liposomes [87–89].

Paclitaxel is a chemotherapeutic that inhibits cell division through promotion of the assembly and stabilization of microtubules. Unfortunately, paclitaxel is highly hydrophobic, and its absorption across the BBB is also poor. To overcome this limitation, paclitaxel has been conjugated to liposomes [90]. Recently, Xin et al. determined the potential of Angiopep-conjugated PEG-PCL nanoparticles loaded with paclitaxel as a dual-targeting drug delivery system in the treatment of glioma. Nanoparticles were conjugated to Angiopep (ANG-NP) for enhanced delivery across the BBB as well as for targeting the tumor via lipoprotein receptor-related protein-mediated endocytosis. Treatment with paclitaxel-loaded ANG-NP resulted in enhanced inhibitory effects in both the antiproliferative and cell apoptosis assay on U87 MG glioma cells. Also, the transport ratios across the BBB model *in vitro* using transwell membrane were significantly increased, and the cell viability of U87 MG glioma cells after crossing the BBB was significantly decreased by ANG-NP-paclitaxel [91]. Additionally, paclitaxel has been attached to an amphiphilic block copolymer of PEG-poly(lactic acid) (PLA) to form a polymer-drug conjugate. Due to the amphiphilicity of this conjugate, after self-assembling in aqueous medium, the paclitaxel molecule was trapped in the core part of the micelles formed and gets well protected, and the PEG segments constitute the upper part of the micelles, and they remain soluble in water [92]. The PEG-PLA-paclitaxel micelles displayed enhanced inhibition ability to tumor growth as shown by the body weight change, survival time, and tumor image size. This improved therapeutic effect was ascribed to the enhanced permeation and retention effect of the PEG-PLA-paclitaxel micelles. Fluorescent imaging of the brain slice further confirmed that rats treated with blank PEG-PLA micelles and PEG-PLA-paclitaxel micelles can pass the BBB and remain in the brain, which displayed higher cell uptake and stronger inhibition and apoptosis toward glioma cells [91].

Curcumin is a polyphenolic compound derived from the Indian spice turmeric. It has been shown to exert antitumor effects in many different cancer cell lines and animal models either by proapoptotic, antiangiogenic, anti-inflammatory, immunomodulatory, and antimitogenic effects [93–95]. Some potential molecular targets for curcumin include insulin-like growth factor (IGF), serine threonine protein kinase (Akt), mitogen-activated protein kinase (MAPK), signal transducer, the activator of transcription 3 (STAT3), nuclear factor kappa *β* (NF*κβ*), and Notch [96, 97]. These pathways are all thought to be active in malignant brain tumors, raising the possibility that curcumin could be effective in treating these diseases [98, 99]. Lim et al. used nanoparticle-encapsulated curcumin to treat medulloblastoma and glioblastoma cells, causing a dose-dependent decrease in growth of multiple brain tumor cell cultures. The reduction in viable neoplastic cells was associated with a combination of G2/M arrest and apoptotic induction [100]. Also, curcumin has been used in a spherical core-shell nanostructure formed by amphiphilic methoxy polyethylene glycol-poly(caprolactone) (mPEG-PCL) block copolymers and was effectively transported and delivered into C6 glioma cells through endocytosis of the nanoparticles and localized around the nuclei in the cytoplasm. *In vitro* studies proved that the cytotoxicity of these nanoconjugates would be result of a pro-apoptotic effect against rat C6 glioma cell line in a dose-dependent manner [101].

Celecoxib, a cyclo-oxygenase- (COX-) 2 inhibitor, has been reported to mediate growth inhibitory effects and to induce apoptosis in various cancer cell lines [102]. Celecoxib has been conjugated to poly-D,L-lactide-co-glycolide (PLGA) and tested in glioma finding that the celecoxib recovered in the nanoparticles showed similar antitumor activity against U87MG cells and C6 cells in a dose-dependent manner. These results show that PLGA nanoparticles incorporating celecoxib are promising candidates for antitumor drug delivery [103].

Although doxorubicin (DOX) has not been used as treatment in brain tumors, because it has poor distribution and limited penetration, it is one of the most likely candidates for CNS chemotherapy [104]. Here, we describe some studies where liposomes were used as carriers of DOX. The liposomal encapsulation of DOX using polyethylene glycol (PEG) liposomes has shown a long circulation time in plasma, reduced cardiac toxicity, and improved penetration of DOX across the BBB by leading to increased efficacy of the distribution and accumulation into tumors [105]. The first studies compared the accumulation between free doxorubicin and PEG-liposome encapsulated in glioma C6 cells models, showing an increase in their uptake and accumulation by glioma cells compared to conventional liposomes or free doxorubicin. Also, the incorporation of PEG into this liposome membrane allowed a long circulating half-life, slow plasma clearance, and a reduced volume of distribution [106].

Nanoconjugates coupled with liposomes could be a new treatment of gliomas, because they increase both the uptake of and specificity to glioma cells. The chlorotoxin (CTx) is a scorpion-derived peptide, which binds with high specificity to glioma cell surface as a specific chloride channel and matrix metalloproteinase-2 blocker. It was firstly applied to establish the CTx-modified doxorubicin- (DOX-)loaded liposome delivery system for targeting brain glioma and improving the anticancer efficacy. Recently, it has been developed in BALB/c mice-bearing U87 tumor xenografts, a novel liposome system with a uniform distribution, high encapsulation efficiency, and adequate loading capacity of both fluorescent probe and DOX. The biodistribution of DOX-loaded liposomes by body imaging and antiglioma pharmacodynamics were studied finding that CTx-modified liposomes were drastically accumulated in subcutaneous and intracranial tumors, showing higher tumor growth inhibition and lower blood toxicity in the armpit tumor model. *In vivo* results exhibited good correlation of glioma targeting of the CTx-modified liposomes, with the CTx as the targeting ligand [107].

In order to increase the uptake of these nanoparticles, specific ligands were coupled to the distal ends of the PEG chains to increase their uptake through receptor-mediated targeting while maintaining PEG stability. The membrane transferrin receptor (Tr) mediated endocytosis or internalization of the complex of transferrin bound iron, and the transferrin receptor is the major route of cellular iron uptake. This efficient cellular uptake pathway has been exploited for the site-specific delivery not only of anticancer drugs and proteins but also of therapeutic genes into proliferating malignant cells that overexpress the transferrin receptors [108, 109]. Studies have shown that PEG liposomes coupled to transferrin are able to achieve preferential receptor-mediated targeting of C6 glioma *in vitro* [110, 111]. On the other hand, lactoferrin (Lf) and the procationic liposomes (PCLs) have been conjugated to develop DOX-loaded Lf-PCL (DOX-Lf-PCL) nanoparticles. In primary culture and glioma cell C6 model, DOX-Lf-PCLs showed significantly higher uptake, and their *in vivo* systemic administration increases the accumulation of Lf-PCLs in the brain [87]. These studies suggested that nanoconjugates and Lf-PCLs were available for brain drug delivery representing potential future clinical application.

## 4. Nanocrystals

Nanocrystals are aggregates of molecules that can be combined into a crystalline form of the drug surrounded by a thin coating of surfactant. They have extensive uses in materials research, chemical engineering, and as quantum dots for biological imaging; there is no carrier material as in polymeric nanoparticles [112–114]. Nanocrystalline species may be prepared from a hydrophobic compound and coated with a thin amphiphilic layer. It has been demonstrated that the size and shape of nanocrystals play an important role in their biological activity [115].

Some nanocrystals have shown to inhibit the proliferation in different cancer cells lines [116]. Silver nanoparticles (Ag-NPs) have recently been the focus of intense research due to their capacity to induce the expression of genes associated with cell cycle progression, DNA damage, and apoptosis in human cells at noncytotoxic doses [117]. The toxicity of starch-coated AgNPs have been studied in normal human lung fibroblast cells (IMR-90) and human glioblastoma cells (U251); uptake of AgNPs is predominantly done by endocytosis and partly adhered to membranous surfaces. Once inside, AgNPs show a uniform intracellular distribution of both cytoplasm and nucleus. The accumulation of these particles causes DNA damage and reduces cellular ATP content, causing damages in mitochondria and increasing the production of reactive oxygen species (ROS) in a dose-dependent manner in glioma cells. Also, it has been proposed that AgNPs can induce DNA damage leading to cell cycle arrested in G2/M phase and enhancing the apoptosis rate of cancer cells [118–121].

The combination of AgNPs with magnetic nanoparticles hyperthermia (MNPH) treatment has been used as treatment in glioma model. AgNPs had significant effect on enhancing thermoinduced killing *in vitro*. In the glioma-bearing rat model, AgNPs combined with MNPH enhance Bax (Bcl-2–associated X protein) expression in cancer cells, which was correlated with cell apoptosis induction. The mechanism of thermosensitization by AgNPs might be related to the release of Ag^+^ cation from the silver nanostructures inside cells. Ag^+^ cation has the ability to capture electrons and thus functions as an oxidative agent [18, 122].

Based on thermodynamic constraints, metallic Ag cores have been modified with (in)organic ligands allowing the synthesis of protein-conjugated Ag_2_S nanoparticles that increase physical and chemical stability [123]. Recently, Wang et al. observed cell death that might result from the interaction between mitochondria proteins and Ag^+^ released from nanocrystals, which were predominantly endocytosed, and partly adhered to the membrane surface [78]. Even when high dosages can be achieved with nanocrystals and poorly soluble drugs can be formulated to increase their bioavailability via treatment with an appropriate coating layer, studies about the stability of nanocrystals and the cytotoxicity of these nanoparticles, with respect to their size and shape, are needed in order to advance in nanotechnology for tumor treatment, cure and to predict the possible toxic side effects on the body [18, 78].

### 4.1. Nanotubes

Self-assembling sheets of atoms arranged in tubes are defined as nanotubes. They may be organic or inorganic in composition and can be produced as single- or multiwalled structures. They have large internal volumes, and the external surface can be easily functionalized. While they are potentially promising for pharmaceutical applications, human tolerance of these compounds remains unknown, toxicity reports are conflicting, and extensive researches regarding the biocompatibility and toxicity of nanotubes are needed [124]. Carbon nanotubes (CNTs) are formed by rolling sheets of graphite-like carbon with hollow tubes. They are categorized based on the number of carbon layers assembled together: single-walled (SW), double-walled (DW), and multiwalled (MW). The diameters vary according to the number of layers: 0.4–2 nm for SWNTs, 1–3.5 nm for DWNTs, and 2–100 nm for MWCNTs [125]. The length of these tubes can be extended to tens of micrometers and is dependent on the method of production.

As in nanovectors, CNTs have the advantage of providing a versatile, biodegradable, and nonimmunogenic delivery alternative to viral vectors for molecular therapy or immunotherapy as direct delivery of antigens to antigen presenting cells (APCs) or microglia in the central nervous system [126]. Kateb et al. evaluated the efficacy of multiwalled carbon nanotubes (MWCNTs) as potential nanovectors for delivery of macromolecules into microglia (MG) using the cell line BV2 (a microglia cell line) to determine the capacity to uptake MWCNTs by BV2 cells *in vitro*, demonstrating the ability of BV2 cells to more efficiently internalize MWCNTs as compared to glioma cells without any significant signs of cytotoxicity. They were able to visualize ingestion of MWCNTs into MG, cytotoxicity, and loading capacity of MWCNTs under normal culture conditions, suggesting that MWCNTs could be used as a novel, nontoxic, and biodegradable nanovehicles for targeted therapy in brain tumors.

On the other hand, this group also analyzed the internalization of these nanotubes in an intracranial glioma model and characterized some changes in tumor cytokine production following intratumoral injection of MWCNTs in GL261 murine glioma model. Authors demonstrated that MWCNTs were preferentially detected in tumor macrophages (MPs), and to a lesser extent in MG. In addition to MG and MP, a small fraction of glioma cells, which are not typically capable of phagocytosis, also became positive for MWCNTs; FACS and quantitative RT-PCR were performed to analyze the inflammatory response and cytokine profile. A transient influx of MP was seen in both normal brain and GL261 gliomas in response to MWCNTs; whereas no significant change in cytokine expression was noted in normal group [127]. They concluded that CNTs can potentially be used as a nanovector delivery system to modulate MP function in tumors.

### 4.2. Inorganic Nanoparticles

Ceramic nanoparticles are typically composed of inorganic compounds such as silica or alumina. Originally used with silica-based materials [128], this approach was extended to organosilicates [129], transition metal oxides [130], metalloid [131], and metal sulfides [132] to produce a myriad of nanostructures with a characteristic size, shape, and porosity. Generally, inorganic nanoparticles may be engineered to evade the reticuloendothelial system by varying their size and surface composition. Moreover, the nanoparticle structure is porous, and it provides a physical encasement to protect an entrapped molecular payload from degradation or denaturation. Mesoporous silica materials contain a complex “worm-like” network of channels throughout the interior of the solid nanoparticles. It is relatively easy to modify the surfaces of these particles with unique functionalities via a variety of chemical transformations. Several functional groups can be introduced onto the surface of inorganic nanoparticles, ranging from saturated and unsaturated hydrocarbons to carboxylic acids, thiols, amines, and alcohols. Inorganic nanoparticles are relatively stable over broad ranges of temperature and pH, yet their lack of biodegradation and slow dissolution raises safety questions, especially for long term administration [133, 134].

### 4.3. Dendrimers

Dendrimers are polymer-based macromolecules formed from monomeric or oligomeric units, such that each layer of branching units doubles or triples the number of peripheral groups. These structures are considered as one of the most promising polymer architectures in biomedical applications in recent years [135, 136]. Such structures are highly branched, multigenerational nanoparticles consisting of exterior end groups that can be functionalized [137–139]. Examples included the encapsulation of therapeutic agents inside the dendrimers and attachment of drugs, targeting moieties and functional groups on the surface of them by covalent bounding or physical absorbing, which afforded the possibility to produce the desired multifunctional nanocarriers for drug delivery.

The avoid area within dendrimer and the extent of its branching, the size control, and its facility of modification and preparation offer great potential for drug delivery. Generally, they have a symmetrical structure, with the potential to create an isolated “active site” core area through chemical functionalization. The modification of the degree of branching may allow for encapsulation of a molecule within this structure [140]. For instance, a dendrimer may become water soluble when its end groups are functionalized with hydrophilic groups, such as carboxylic acids. Thus, water-soluble dendrimers may be designed with internal hydrophobicity, suitable for the incorporation of a hydrophobic drug. The frequently used genetic transfection agent *polyfect* consists of dendrimer molecules radiating from a central core. Amino groups at the terminal ends of the dendrimer branches are positively charged at physiological pH, therefore interacting with the negatively charged phosphate groups of nucleic acids [141]. However, dendrimers require further improvements in cytotoxicity profiles, biocompatibility, and biodistribution into the body.

Drug carriers such as dendrimers have been used for therapeutic purposes in the treatment of gliomas. These nanomaterials were conjugated to D-glucosamine as the ligand for enhancing their permeability across BBB and tumor targeting. The efficacy of methotrexate- (MTX-)loaded dendrimers was established against U87 MG and U343 MGa cells. Permeability of rhodamine-labeled dendrimers and MTX-loaded dendrimers across an *in vitro* BBB model and their distribution into vascular human glioma tumor spheroids were also studied. Glycosylated dendrimers were found to be endocytosed in significantly higher amounts than nonglucosylated dendrimers by the cell lines mentioned above. These MTX-loaded dendrimers were also able to kill even MTX-resistant cells highlighting their ability to overcome MTX resistance. In addition, the amount of MTX transported across BBB was three to five times more after loading in the dendrimers. Glycosylation further increased the cumulative permeation of dendrimers across BBB and hence increased the amount of MTX available across it. These results shown that glucosamine not only can be used as an effective ligand for targeting glial tumors but also enhanced their permeability across BBB [142].

Furthermore, the poly(amidoamine) (PAMAM) dendrimer was employed as a carrier to codeliver antisense-miR-21 oligonucleotide (as-miR-21) and 5-fluorouracil (5-FU) to achieve delivery of as-miR-21 to human glioblastoma cells and enhance the cytotoxicity of 5-FU antisense therapy. PAMAM could be simultaneously loaded with 5-FU and as-miR-21, forming a complex smaller than 100 nm in diameter. Both the chemotherapeutant and as-miR-21 could be efficiently introduced into tumor cells. The codelivery of as-miR-21 significantly improved the cytotoxicity of 5-FU and dramatically increased the apoptosis of U251 cells, while the migration ability of the tumor cells was decreased. These results suggest that the codelivery system may have important clinical applications in the treatment of miR-21-overexpressing glioblastoma [143].

## 5. Antibodies Conjugated to Nanoparticles

Tumor-specific targeting using achievements of nanotechnology is a mainstay of increasing efficacy of antitumor drugs. One of the most significant advances in tumor-targeted therapy is the surface modification of nanoparticles with monoclonal antibodies (mAbs) alone or in combination with antineoplastic drugs in cancer therapy [144]. Another important advantage of this technology is the possibility of masking the unfavorable physicochemical characteristics of the incorporated molecule. In particular, the treatment of brain tumors takes advantage of this characteristics due to efficient and specific brain delivery of the anticancer drugs [145]. These different strategies can be exploited for a variety of biomedical applications such as cancer immunotherapy that manipulate the immune system for therapeutic benefits and minimize adverse effects [146]. 

In order to improve direct tumor targeting and to avoid the damage of nontumor cells Fujita et al. [147] synthesized a new polycefin variant conjugated to two monoclonal antibodies of different specificities in a promising drug carrier poly(*β*-l-malic acid) (PMLA) polymer, natural product of *Physarum polycephalum* [148] that is used as a carrier matrix of biopharmaceuticals with some advantages such as lack of toxicity *in vitro* and *in vivo*, nonimmunogenicity, biodegradability, stability in the bloodstream, and easy cellular uptake [149–152]. Also, they studied the drug accumulation in glioma-bearing animals finding that the polycefin variant with the combination of mouse anti-TfR [153, 154] and human tumor-specific antibody 2C5 [155, 156] provides the most efficient drug delivery route through mouse endothelial system and into implanted human brain tumor cells. It was not achieved by variants with single mAbs or devoid of antibodies. The presence of two or more different antibodies at the same time on drug delivery systems, especially on polycefin variants, may be important for future specific drug delivery and therapeutic efficacy in tumor treatment.

Another interesting approach is the use of immunoliposomes, which are antibodies conjugated to the liposomes using the antibody motif of protein A (ZZ) as an adaptor. Feng et al. [157] used the immunoliposomes to deliver sodium borocaptate (BSH) encapsulated in liposomes composed of nickel lipid (a lipid derivatized with a nickel-chelating head group) and antiepidermal growth factor receptor (EGFR); antibodies were conjugated to the liposomes using the antibody affinity motif of protein A (ZZ) as an adaptor into EGFR-overexpressing glioma cells. Immunohistochemical analysis using an anti-BSH monoclonal antibody revealed that BSH was delivered effectively into the cells but not into EGFR-deficient glioma or primary astrocytes. In an animal model of brain tumors, both the liposomes and the BSH were only observed in the tumor. Moreover, enriched boron or ^10^B conjugated with anti-EGFR antibodies by ZZ-His provides a selective delivery system into glioma cells, and this was confirmed by inductively coupled plasma-atomic emission spectrometry (ICP-AES) both *in vitro* and *in vivo* [157].

### 5.1. Solid Lipid Nanoparticles

Solid lipid nanoparticles are lipid-based submicron colloidal carriers. They were initially designed in the early 1990s as a pharmaceutical alternative to liposomes and emulsions. In general, they are more stable than liposomes in biological systems due to their relatively rigid core consisting of hydrophobic lipids that are solid at room and body temperatures, surrounded by a monolayer of phospholipids [158]. These aggregates are further stabilized by the inclusion of high levels of surfactants. Because of their facility of biodegradation, they are less toxic than polymer or ceramic nanoparticles. Also, they have controllable pharmacokinetic parameters and can be engineered with three types of hydrophobic core designs: a homogenous matrix, a drug-enriched shell, or a drug-enriched core. It has been demonstrated that the compound payload can leave the hydrophobic core at warmer temperatures. Conversely, the compound payload enters the hydrophobic core at lower temperatures. These principles are used to load and unload solid lipid nanoparticles for the delivery of therapeutic agents, taking advantage of recent techniques to selectively produce hypo- and hyperthermia. These nanoparticles can be used to deliver drugs orally, topically, or via inhalation.

Recently, Kuo and Liang used innovative catanionic solid lipid nanoparticles (CASLNs) prepared in microemulsions carrying carmustine (BCNU) (BCNU-CASLNs) that were grafted with antiepithelial growth factor receptor (EGFR) (anti-EGFR/BCNU-CASLNs) and applied to inhibit the propagation of human brain malignant glioblastomas cells due to gliomas normally express certain types of growth factor receptor. The catanionic surfactants (1 Mm) yielded the smallest particle size of BCNUCASLNs and the largest entrapment efficiency of BCNU with a moderate toxicity to human brain-microvascular endothelial cell and a tolerable expression of TNF-*α*. Thereby, anti-EGFR/BCNU-CASLNs could have a potential use in anticancer chemotherapy for clinical application [159].

These nanoparticles could be loaded with others types of chemotherapeutics such as doxorubicin (DOX). The use of CASLNs loaded with DOX and grafted with antiepithelial growth factor receptor (EGFR) (anti-EGFR/DOX-CASLNs) suppressed the propagation of malignant U87MG cells. At 1 mM concentrations of these catanionic surfactants conjugated with hexadecyltrimethylammonium bromide and sodium anionic sodium dodecylsulfate, CASLNs entrapped the largest quantity of DOX, concluding that catanionic surfactants at 1 mM and 100% of cacao butter (CB) could be satisfactory conditions for preparing anti-EGFR/DOX-CASLNs to inhibit proliferation of malignant U87MG cells, and the grafted anti-EGFR could substantially enhance the delivery efficiency of DOX to U87MG cells [160].

Above all, these nanoparticles are not used yet in clinical trials against glioblastoma or others types of brain tumors, but this innovative approach can be an effective delivery system with high targeting efficacy against brain tumors due to the great capacity to deliver chemotherapeutic agents and to reduce toxicity.

In Table 2 we are summarized some of the most applied nanoparticles for treatment of gliomas.

## 6. Special Considerations

While it is important to achieve an increased uptake of functional targeting nanoparticles by GBM cells, it is also important to consider the biodistribution of the nanoparticles in blood circulation and liver accumulation, highlighting the importance of controlling ligand loading in order to achieve optimal performance for therapeutic and imaging applications for multivalent nanoparticle-based systems [161]. 

## 7. Perspectives

Nowadays, several research groups are actively trying to combine a variety of functions into NPs as platforms for targeting different immune and tumoral cells and to develop diverse strategies to modulate specific treatments. A major effort toward successful NP-based therapeutics will be needed to avoid extensive and nonspecific immunostimulatory or immunosuppressive reactions to the nanomaterials, once they have been administered into the body, in order to find a right balance between any remaining potential damage and the health and quality of life of patients. This implies the future development of new or adapted methods appropriate to assess new medicinal tools involving NPs. Although many questions still require extensive investigation, the available data suggest that a variety of NPs can be engineered to become part of the next generation of immunomodulatory platforms and treatments [80].

Nanoparticles exploit biological pathways to achieve payload delivery to cellular and intracellular targets, including transport across the BBB. The central nervous system is protected by this barrier which maintains its homeostasis. However, many potential drugs for the treatment of diseases of the central nervous system (CNS) cannot reach the brain in high concentrations. This physical barrier limits the brain uptake of the vast majority of neurotherapeutics and neuroimaging contrast agents [7, 17]. One possibility to deliver drugs to the CNS is the employment of polymeric nanoparticles. Modification of the nanoparticle surface with covalently attached targeting ligands or by coating with certain surfactants had enabled the adsorption of specific plasma proteins. The ability of these carriers to overcome BBB appears to be receptor-mediated endocytosis in brain capillary endothelial cells. The possibility to employ nanoparticles for delivery of proteins and other macromolecules across the BBB suggests that this technology holds great promises for noninvasive therapy of the CNS diseases. Recently, some studies have shown the distribution, pharmacokinetics, and drugs delivery into the brain in rodents and found that nanoparticles greater than approximately 100–150 nm in diameter will tend to accumulate in tumors due to their higher extravasation in comparison with normal vasculature [16, 162]. Rapid advances and emerging technologies in nanoparticles have shown a profound impact on cancer diagnosis, treatment, and monitoring. Now, the interest of researchers is defining physical and chemical characteristics to provide an effective therapy without side effects.

## 8. Conclusion

Studies on the biological composition, administrations, and adverse events of new nanomaterials suited for biomedical applications are important for therapeutic drug delivery and the development of innovative and better treatments [163]. Furthermore, the engineering of the particle backbone structure, size, shape of the nanoparticle surface, and the core itself provides yet another dimension of physical control that can be directed toward an increased strength, increased chemical specificity, or heat resistance. Most polymeric nanoparticles are biodegradable and biocompatible and have been adopted as a preferred method for drug delivery. Since nanoparticles come into direct contact with cellular membranes, their surface properties may determine the mechanism of internalization and intracellular localization [164]. They also exhibit a good potential for surface modification via chemical transformations, provide excellent pharmacokinetic control, and are suitable for the entrapment and delivery of a wide range of therapeutic agents.

The use of nanoparticles could be a good option in diagnosis and treatment of gliomas. Studies suggest that a variety of NPs can be engineered to become part of the next generation of agents delivery and specific treatment on gliomas. The use of a biocompatible system of NPs, conjugates should reduce the toxicity and side effects of systemic drugs administration and therefore improve the quality of life in cancer patients. However, several studies conducted largely on mice have shown undesired side effects such as inflammatory response including substantial lung neutrophil influx and mortality at high doses. In addition, NPs may feasibly represent a useful imaging tool to diagnosis and followup; also, it to be used to assess/monitor efficacy of antiangiogenic or other antitumour treatments, thus improving the clinical management of brain tumours. Nevertheless, additional research is required in multifunctional NPs based drug delivery systems to overcome the problems and understand how nanoparticles interact with biological systems and the environment for effective therapy.

## Figures and Tables

**Table 1:** Some nanoparticles used in diagnosis of glioblastoma multiforme.

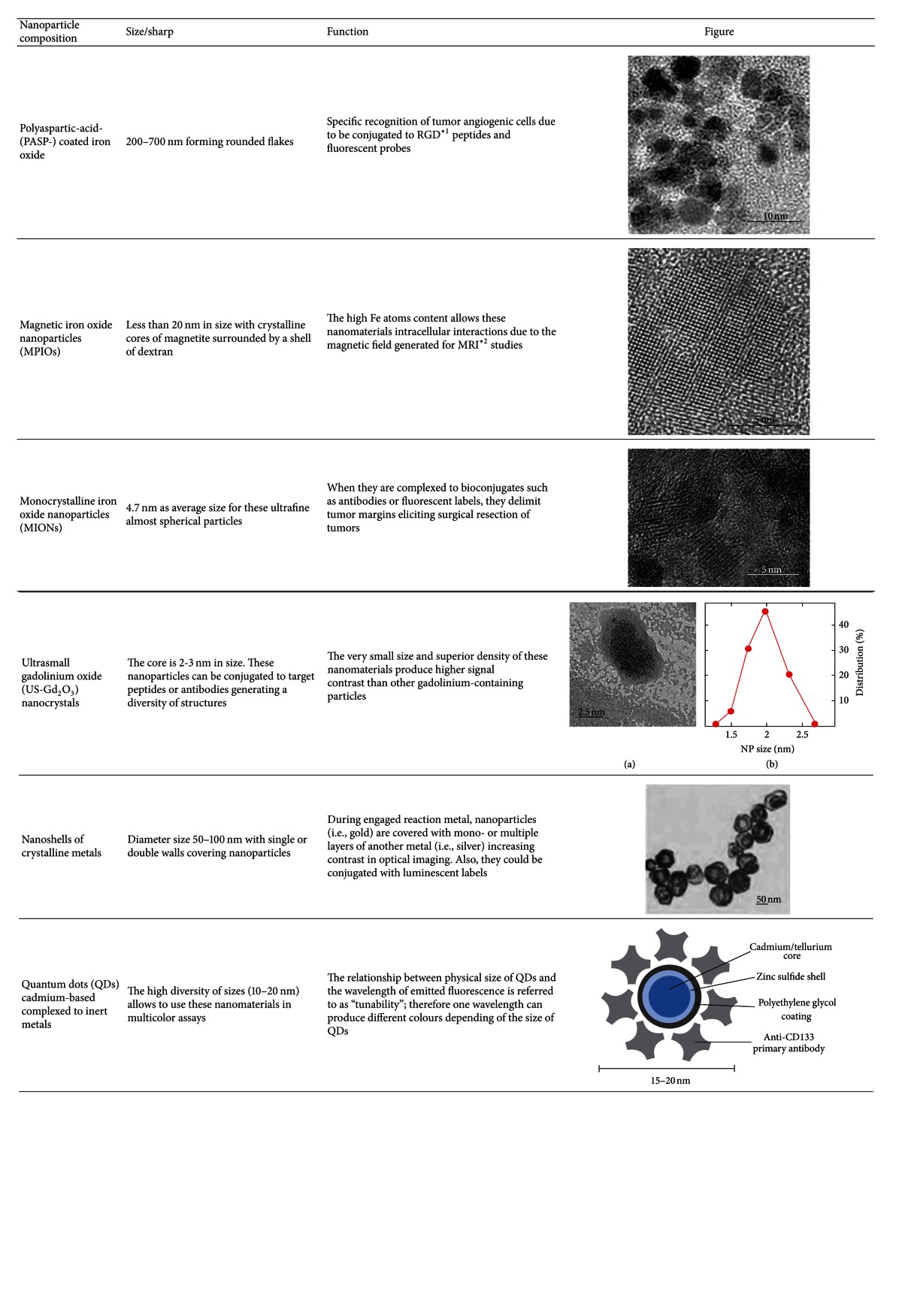

^∗1^RGD: arginine-glycine-aspartic acid sequence.

^∗2^MRI: magnetic resonance imaging.

**Table 2:** Nanoparticles proposed as candidates for the treatment of glioblastoma multiforme.